# The genome sequence of the double-striped pug, *Gymnoscelis rufifasciata *(Haworth, 1809)

**DOI:** 10.12688/wellcomeopenres.17790.1

**Published:** 2022-04-13

**Authors:** Douglas Boyes, University of Oxford and Wytham Woods Genome Acquisition Lab, Darwin Tree of Life Barcoding collective, Wellcome Sanger Institute Tree of Life programme, Wellcome Sanger Institute Scientific Operations: DNA Pipelines collective, Tree of Life Core Informatics collective, Thomas Lewin, Darwin Tree of Life Consortium

**Affiliations:** 1 UK Centre for Ecology and Hydrology, Wallingford, Oxfordshire, UK; 2 Department of Zoology, University of Oxford, Oxford, UK

**Keywords:** Gymnoscelis rufifasciata, double-striped pug, genome sequence, chromosomal, Lepidoptera

## Abstract

We present a genome assembly from an individual female *Gymnoscelis rufifasciata *(the double-striped pug; Arthropoda; Insecta; Lepidoptera; Geometridae). The genome sequence is 352 megabases in span. The majority of the assembly (99.82%) is scaffolded into 32 chromosomal pseudomolecules, with the W and Z sex chromosomes assembled. The mitochondrial genome was also assembled, and is 15.4 kilobases in length.

## Species taxonomy

Eukaryota; Metazoa; Ecdysozoa; Arthropoda; Hexapoda; Insecta; Pterygota; Neoptera; Endopterygota; Lepidoptera; Glossata; Ditrysia; Geometroidea; Geometridae; Larentiinae; Gymnoscelis; *Gymnoscelis rufifasciata* (Haworth, 1809) (NCBI:txid934940).

## Background

The double-striped pug ( *Gymnoscelis rufifasciata*), named after the two dark bands across its wings, is a moth of the family Geometridae. A typical adult’s wingspan is 15 to 19 mm, with forewings varying from light brown to dark reddish-brown, and the intensity of its stripes also variable ( Riley & Prior, 2003; Skinner & Wilson, 2009). A double brooded species, adults are first on the wing in the UK from March to May, although they have been observed as early as January in the mildest winters, and then again from July to August ( Randle *et al*., 2019); they are frequently found in light traps throughout these periods.

*Gymnoscelis rufifasciata* is common across western and central Europe, and while it has long been common in southern England, observations in northern England and Scotland have increased dramatically since the year 2000 ( Fox *et al*., 2021), likely caused by the increasing suitability of the climate due to rising average temperatures. Indeed, *G. rufifasciata* appears to be thriving in the current climate, and its abundance in Britain was reported to have increased 220% from 1986 to 2016 ( Randle *et al*., 2019); its generalist habitat usage, which includes gardens, wasteland, heathland and hedgerows ( Riley & Prior, 2003; Randle *et al*., 2019) is likely to have contributed to its recent success. Its larvae are polyphagous, and favoured food plants include gorse ( *Ulex*), holly ( *Ilex*) and heather ( *Calluna*) ( Riley & Prior, 2003; Skinner & Wilson, 2009).

## Genome sequence report

**Figure 1.**
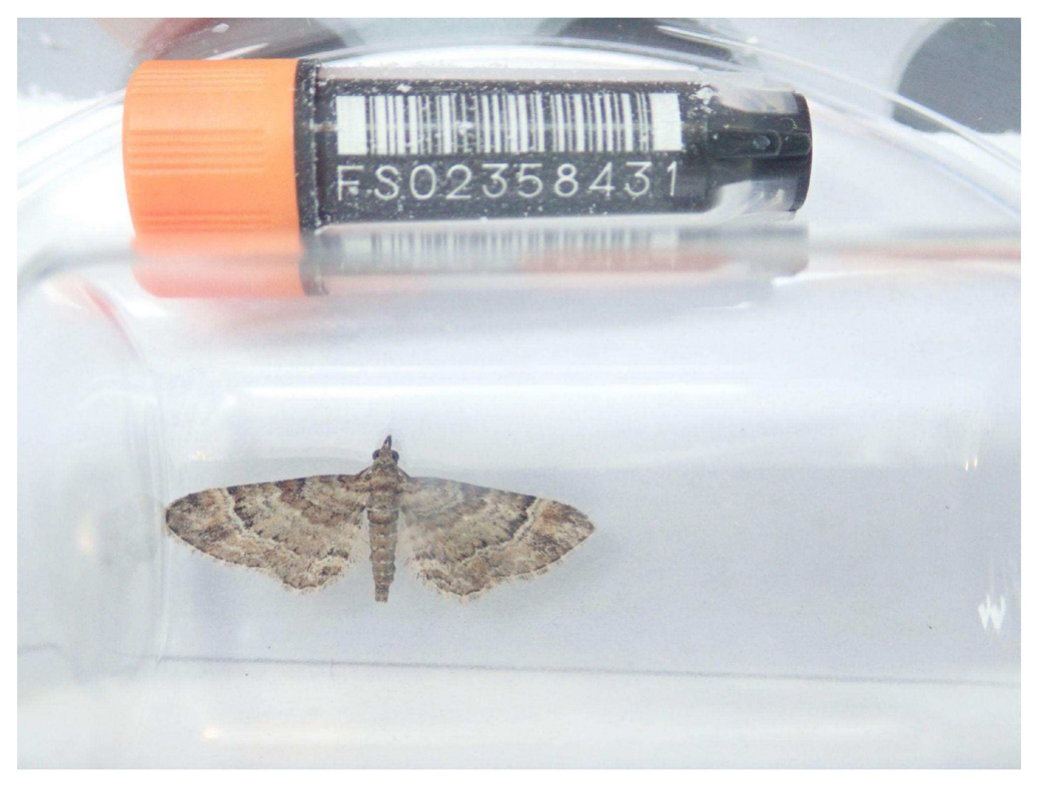
Image of the *Gymnoscelis rufifasciata* (ilGymRufi1) specimen taken prior to preservation and processing. Specimen shown next to FluidX storage tube, 43.9 mm in length.

The genome was sequenced from one female *G. rufifasciata* ( Figure 1) collected from Wytham Woods, Oxfordshire (biological vice-county: Berkshire), UK (latitude 51.765, longitude -1.327). A total of 58-fold coverage in Pacific Biosciences single-molecule long reads and 102-fold coverage in 10X Genomics read clouds were generated. Primary assembly contigs were scaffolded with chromosome conformation Hi-C data. Manual assembly curation corrected 11 missing/misjoins and removed 3 haplotypic duplications, reducing the assembly size by 0.54% and the scaffold number by 19.05%.

**Figure 2.**
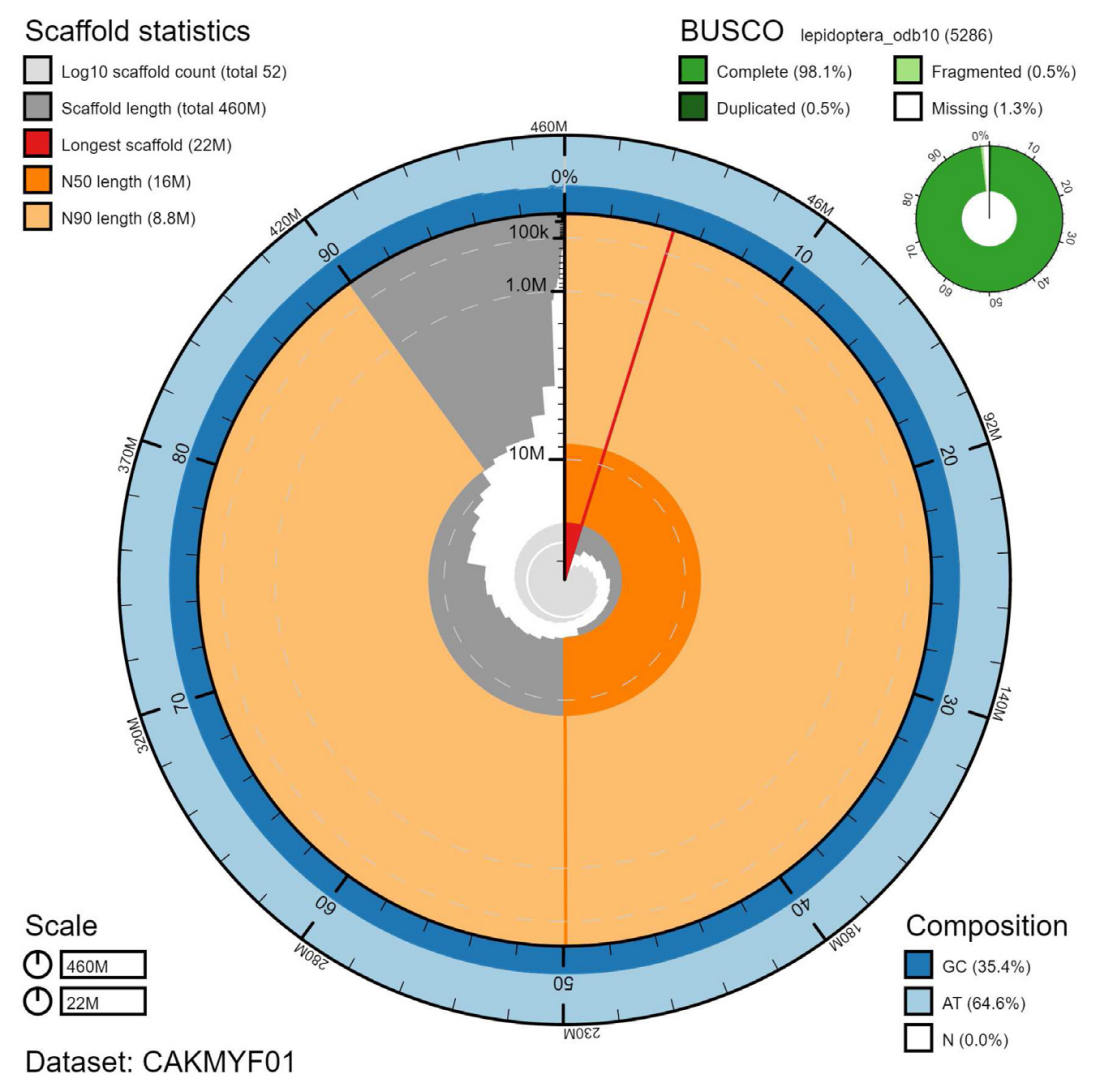
Genome assembly of *Gymnoscelis rufifasciata*, ilGymRufi1.1: metrics. The BlobToolKit Snailplot shows N50 metrics and BUSCO gene completeness. The main plot is divided into 1,000 size-ordered bins around the circumference with each bin representing 0.1% of the 462,009,964 bp assembly. The distribution of scaffold lengths is shown in dark grey with the plot radius scaled to the longest scaffold present in the assembly (22,195,239 bp, shown in red). Orange and pale-orange arcs show the N50 and N90 scaffold ome lengths (15,602,950 and 8,767,853 bp), respectively. The pale grey spiral shows the cumulative scaffold count on a log scale with white scale lines showing successive orders of magnitude. The blue and pale-blue area around the outside of the plot shows the distribution of GC, AT and N percentages in the same bins as the inner plot. A summary of complete, fragmented, duplicated and missing BUSCO genes in the lepidoptera_odb10 set is shown in the top right. An interactive version of this figure is available at https://blobtoolkit.genomehubs.org/view/ilGymRufi1.1/dataset/CAKMYF01/snail.

The final assembly has a total length of 462 Mb in 51 sequence scaffolds with a scaffold N50 of 15.6 Mb ( Table 1). The majority of the assembly sequence (99.82%) was assigned to 32 chromosomal-level scaffolds, representing 30 autosomes (numbered by sequence length), and the W and Z sex chromosomes ( Figure 2– Figure 5; Table 2). The assembly has a BUSCO v5.2.2 ( Manni *et al*., 2021) completeness of 98.1% (single 97.7%, duplicated 0.5%) using the lepidoptera_odb10 reference set. While not fully phased, the assembly deposited is of one haplotype. Contigs corresponding to the second haplotype have also been deposited.

**Table 1.** Genome data for *Gymnoscelis rufifasciata*, ilGymRufi1.1.

*Project accession data*	
Assembly identifier	ilGymRufi1.1
Species	*Gymnoscelis rufifasciata*
Specimen	ilGymRufi1 (genome assembly); ilGymRufi2 (Hi-C)
NCBI taxonomy ID	NCBI:txid934940
BioProject	PRJEB48374
BioSample ID	SAMEA7519910
Isolate information	Female, whole organism (ilGymRufi1 genome assembly), unknown sex, whole organism (Hi-C, ilGymRufi2)
*Raw data accessions*	
PacificBiosciences SEQUEL II	ERR7221644
10X Genomics Illumina	ERR7220453-ERR7220456
Hi-C Illumina	ERR7220457
*Genome assembly*	
Assembly accession	GCA_929108375.1
Accession of alternate haplotype	GCA_929108405.1
Span (Mb)	462
Number of contigs	62
Contig N50 length (Mb)	15.6
Number of scaffolds	51
Scaffold N50 length (Mb)	15.6
Longest scaffold (Mb)	18.8
BUSCO * genome score	C:98.1%[S:97.7%,D:0.5%], F:0.5%,M:1.3%,n:5286

*BUSCO scores based on the lepidoptera_odb10 BUSCO set using v5.2.2. C= complete [S= single copy, D=duplicated], F=fragmented, M=missing, n=number of orthologues in comparison. A full set of BUSCO scores is available at https://blobtoolkit.genomehubs.org/view/ilGymRufi1.1/dataset/CAKMYF01/busco.

**Figure 3.**
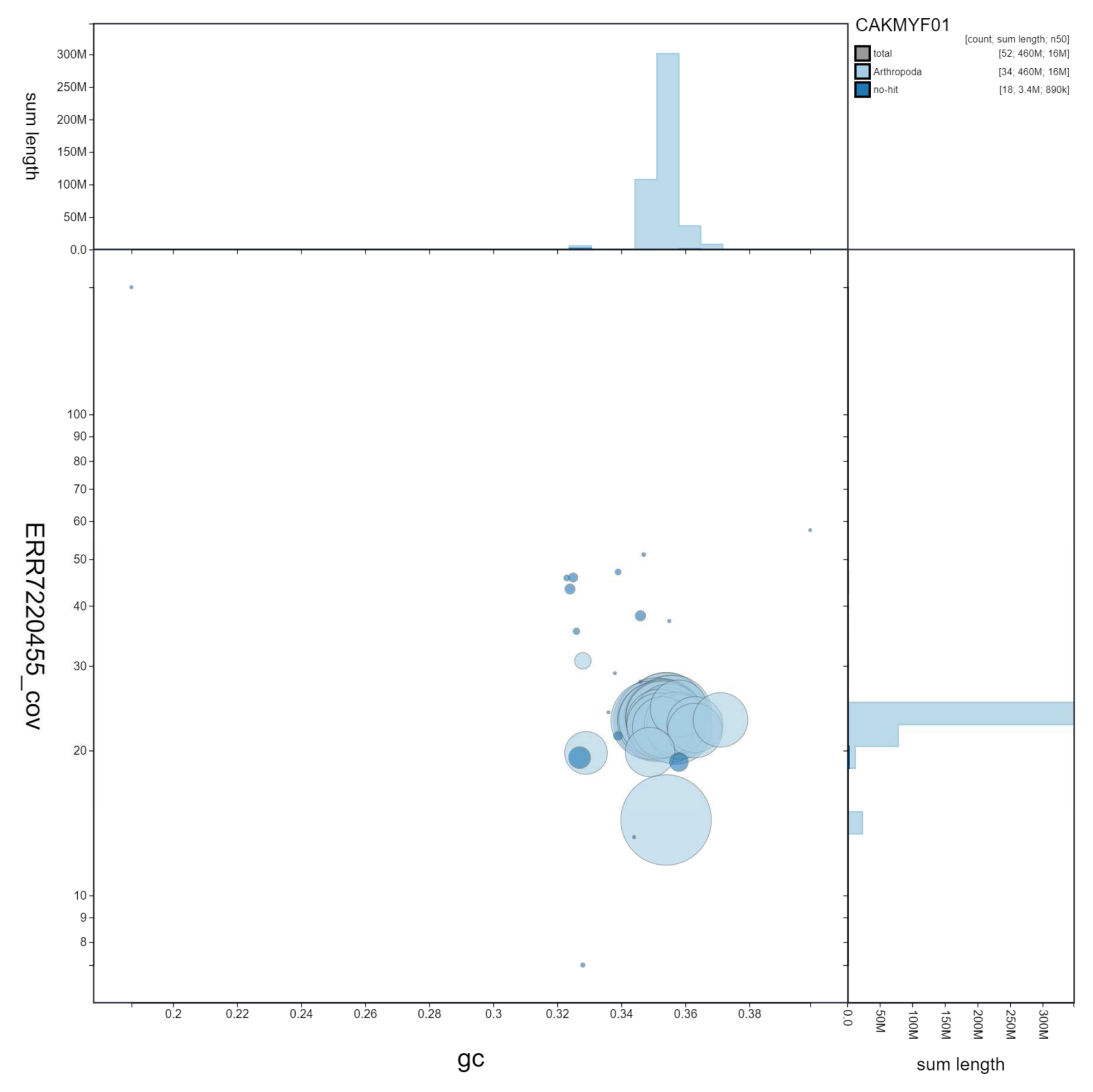
Genome assembly of *Gymnoscelis rufifasciata*, ilGymRufi1.1: GC coverage. BlobToolKit GC-coverage plot. Scaffolds are coloured by phylum. Circles are sized in proportion to scaffold length Histograms show the distribution of scaffold length sum along each axis. An interactive version of this figure is available at https://blobtoolkit.genomehubs.org/view/ilGymRufi1.1/dataset/CAKMYF01/blob.

**Figure 4.**
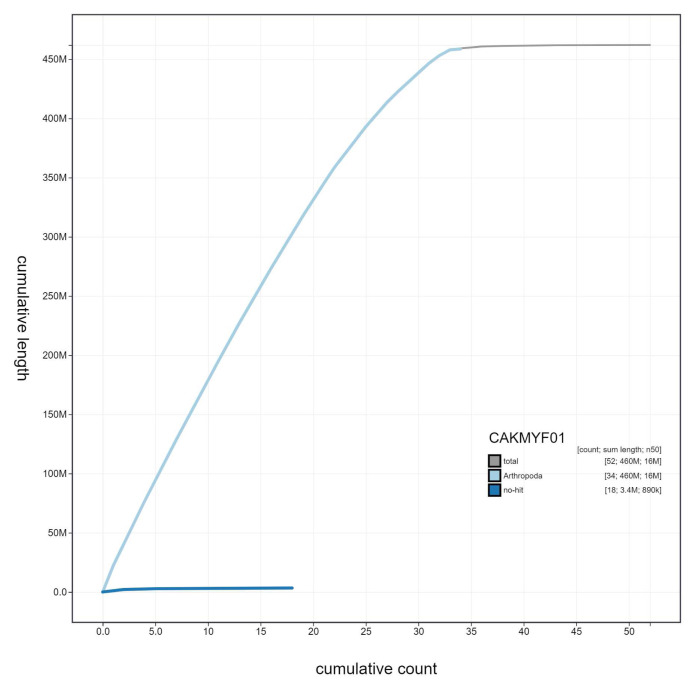
Genome assembly of *Gymnoscelis rufifasciata*, ilGymRufi1.1: cumulative sequence. BlobToolKit cumulative sequence plot. The grey line shows cumulative length for all scaffolds. Coloured lines show cumulative lengths of scaffolds assigned to each phylum using the buscogenes taxrule. An interactive version of this figure is available at https://blobtoolkit.genomehubs.org/view/ilGymRufi1.1/dataset/CAKMYF01/cumulative.

**Figure 5.**
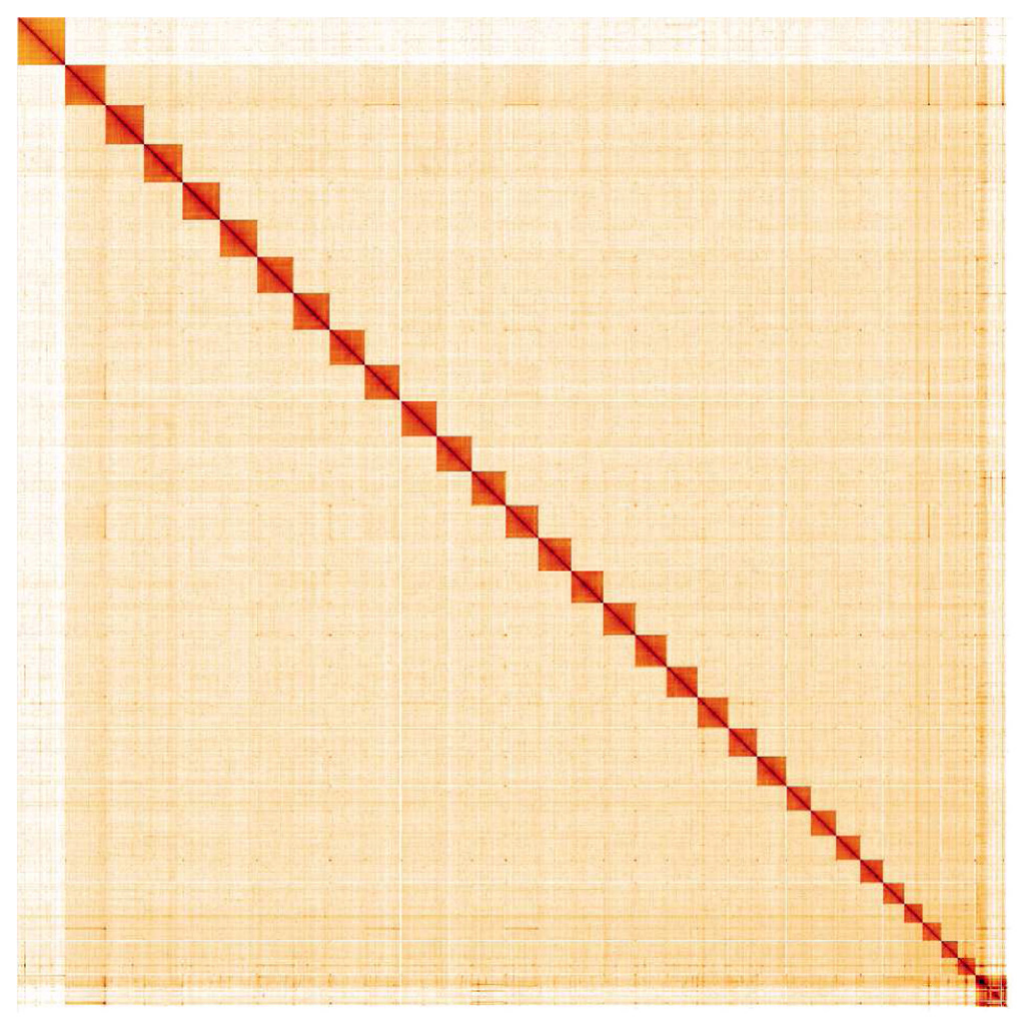
Genome assembly of *Gymnoscelis rufifasciata*, ilGymRufi1.1: Hi-C contact map. Hi-C contact map of the ilGymRufi1.1 assembly, visualised in HiGlass. Chromosomes are shown in order of size from left to right and top to bottom. An interactive version of this map is available here.

**Table 2.** Chromosomal pseudomolecules in the genome assembly of *Gymnoscelis rufifasciata*, ilGymRufi1.1.

INSDC accession	Chromosome	Size (Mb)	GC%
OV815302.1	1	18.83	35.1
OV815303.1	2	18.12	35.4
OV815304.1	3	17.86	35.4
OV815305.1	4	17.46	35.5
OV815306.1	5	17.34	35.0
OV815307.1	6	16.92	35.3
OV815308.1	7	16.84	35.6
OV815309.1	8	16.61	35.1
OV815310.1	9	16.56	34.9
OV815311.1	10	16.46	35.3
OV815312.1	11	16.42	35.1
OV815313.1	12	15.73	35.4
OV815314.1	13	15.60	35.3
OV815315.1	14	15.38	35.1
OV815316.1	15	15.01	35.3
OV815317.1	16	14.84	35.5
OV815318.1	17	14.76	35.3
OV815320.1	18	14.39	35.7
OV815321.1	19	14.02	35.4
OV815322.1	20	13.58	35.5
OV815323.1	21	13.48	35.5
OV815324.1	22	11.75	35.2
OV815325.1	23	11.66	35.2
OV815326.1	24	11.20	35.8
OV815327.1	25	10.47	35.7
OV815328.1	26	9.95	35.3
OV815329.1	27	8.77	35.8
OV815330.1	28	8.40	36.3
OV815331.1	29	8.01	36.3
OV815332.1	30	7.98	37.1
OV815319.1	W	6.55	34.9
OV815301.1	Z	22.20	35.4
OV815333.1	MT	0.02	18.9
-	Unplaced	8.85	33.2

## Methods

### Sample acquisition, DNA extraction and sequencing

A single female *G. rufifasciata* (ilGymRufi1) and a second *G. rufifasciata* of unknown sex (ilGymRufi2) were collected from Wytham Woods, Oxfordshire (biological vice-county: Berkshire), UK (ilGymRufi1: latitude 51.765, longitude -1.327; ilGymRufi2: latitude 51.772, longitude -1.338) by Douglas Boyes, UKCEH, using a light trap in woodland. The sample was identified by the same individual, and preserved on dry ice.

DNA was extracted from whole organism tissue at the Wellcome Sanger Institute (WSI) Scientific Operations core from the whole organism using the Qiagen MagAttract HMW DNA kit, according to the manufacturer’s instructions. Pacific Biosciences HiFi circular consensus and 10X Genomics Chromium read cloud sequencing libraries were constructed according to the manufacturers’ instructions. Sequencing was performed by the Scientific Operations core at the Wellcome Sanger Institute on Pacific Biosciences SEQUEL II (HiFi) and Illumina HiSeq X (10X) instruments. Hi-C data were generated from head tissue of ilGymRufi2 using the Arima Hi-C+ kit and sequenced on an Illumina NovaSeq 6000 instrument.

### Genome assembly

Assembly was carried out with Hifiasm ( Cheng *et al*., 2021); haplotypic duplication was identified and removed with purge_dups ( Guan *et al*., 2020). One round of polishing was performed by aligning 10X Genomics read data to the assembly with longranger align, calling variants with freebayes ( Garrison & Marth, 2012). The assembly was then scaffolded with Hi-C data ( Rao *et al*., 2014) using SALSA2 ( Ghurye *et al*., 2019). The assembly was checked for contamination as described previously ( Howe *et al*., 2021). Manual curation ( Howe *et al*., 2021) was performed using HiGlass ( Kerpedjiev *et al*., 2018) and Pretext. The mitochondrial genome was assembled using MitoHiFi ( Uliano-Silva *et al*., 2021), which performs annotation using MitoFinder ( Allio *et al*., 2020). The genome was analysed and BUSCO scores generated within the BlobToolKit environment ( Challis *et al*., 2020). Table 3 contains a list of all software tool versions used, where appropriate.

**Table 3.** Software tools used.

Software tool	Version	Source
Hifiasm	0.15.3	Cheng *et al*., 2021
purge_dups	1.2.3	Guan *et al*., 2020
SALSA	2.2	Ghurye *et al*., 2019
longranger align	2.2.2	https://support.10xgenomics. com/genome-exome/software/ pipelines/latest/advanced/other- pipelines
freebayes	1.3.1-17-gaa2ace8	Garrison & Marth, 2012
MitoHiFi	2.0	Uliano-Silva *et al*., 2021
HiGlass	1.11.6	Kerpedjiev *et al*., 2018
PretextView	0.2.x	https://github.com/wtsi-hpag/PretextView
BlobToolKit	3.0.5	Challis *et al*., 2020

### Ethics/compliance issues

The materials that have contributed to this genome note have been supplied by a Darwin Tree of Life Partner. The submission of materials by a Darwin Tree of Life Partner is subject to the Darwin Tree of Life Project Sampling Code of Practice. By agreeing with and signing up to the Sampling Code of Practice, the Darwin Tree of Life Partner agrees they will meet the legal and ethical requirements and standards set out within this document in respect of all samples acquired for, and supplied to, the Darwin Tree of Life Project. Each transfer of samples is further undertaken according to a Research Collaboration Agreement or Material Transfer Agreement entered into by the Darwin Tree of Life Partner, Genome Research Limited (operating as the Wellcome Sanger Institute), and in some circumstances other Darwin Tree of Life collaborators.

## Data availability

European Nucleotide Archive: Gymnoscelis rufifasciata (double-striped pug). Accession number PRJEB48374; https://identifiers.org/ena.embl/PRJEB48374.

The genome sequence is released openly for reuse. The *G. rufifasciata* genome sequencing initiative is part of the Darwin Tree of Life (DToL) project. All raw sequence data and the assembly have been deposited in INSDC databases. The genome will be annotated and presented through the Ensembl pipeline at the European Bioinformatics Institute. Raw data and assembly accession identifiers are reported in Table 1.

## Author information

Members of the University of Oxford and Wytham Woods Genome Acquisition Lab are listed here: https://doi.org/10.5281/zenodo.5746938.

Members of the Darwin Tree of Life Barcoding collective are listed here: https://doi.org/10.5281/zenodo.5744972.

Members of the Wellcome Sanger Institute Tree of Life programme are listed here: https://doi.org/10.5281/zenodo.6125027.

Members of Wellcome Sanger Institute Scientific Operations: DNA Pipelines collective are listed here: https://doi.org/10.5281/zenodo.5746904.

Members of the Tree of Life Core Informatics collective are listed here: https://doi.org/10.5281/zenodo.6125046.

Members of the Darwin Tree of Life Consortium are listed here: https://doi.org/10.5281/zenodo.5638618.

## References

[ref-1] Allio R Schomaker-Bastos A Romiguier J : MitoFinder: Efficient Automated Large-Scale Extraction of Mitogenomic Data in Target Enrichment Phylogenomics. *Mol Ecol Resour.* 2020;20(4):892–905. 10.1111/1755-0998.13160 32243090 PMC7497042

[ref-2] Challis R Richards E Rajan J : BlobToolKit - Interactive Quality Assessment of Genome Assemblies. *G3 (Bethesda).* 2020;10(4):1361–74. 10.1534/g3.119.400908 32071071 PMC7144090

[ref-3] Cheng H Concepcion GT Feng X : Haplotype-Resolved *de Novo* Assembly Using Phased Assembly Graphs with Hifiasm. *Nat Methods.* 2021;18(2):170–75. 10.1038/s41592-020-01056-5 33526886 PMC7961889

[ref-4] Fox R Dennis EB Harrower CA : The State of Britain’s Larger Moths 2021. 2021 March, 44. Reference Source

[ref-5] Garrison E Marth G : Haplotype-Based Variant Detection from Short-Read Sequencing. arXiv: 1207.3907,2012. 10.48550/arXiv.1207.3907

[ref-6] Ghurye J Rhie A Walenz BP : Integrating Hi-C Links with Assembly Graphs for Chromosome-Scale Assembly. *PLoS Comput Biol.* 2019;15(8):e1007273. 10.1371/journal.pcbi.1007273 31433799 PMC6719893

[ref-7] Guan D McCarthy SA Wood J : Identifying and Removing Haplotypic Duplication in Primary Genome Assemblies. *Bioinformatics.* 2020;36(9):2896–98. 10.1093/bioinformatics/btaa025 31971576 PMC7203741

[ref-8] Howe K Chow W Collins J : Significantly Improving the Quality of Genome Assemblies through Curation. *GigaScience.* 2021;10(1):giaa153. 10.1093/gigascience/giaa153 33420778 PMC7794651

[ref-9] Kerpedjiev P Abdennur N Lekschas F : HiGlass: Web-Based Visual Exploration and Analysis of Genome Interaction Maps. *Genome Biol.* 2018;19(1):125. 10.1186/s13059-018-1486-1 30143029 PMC6109259

[ref-10] Manni M Berkeley MR Seppey M : BUSCO Update: Novel and Streamlined Workflows along with Broader and Deeper Phylogenetic Coverage for Scoring of Eukaryotic, Prokaryotic, and Viral Genomes. *Mol Biol Evol.* 2021;38(10):4647–54. 10.1093/molbev/msab199 34320186 PMC8476166

[ref-11] Randle Z Evans-Hill LJ Parsons MS : Atlas of Britain and Ireland’s Larger Moths. Pisces Publications, Newbury.2019. Reference Source

[ref-12] Rao SSP Huntley MH Durand NC : A 3D Map of the Human Genome at Kilobase Resolution Reveals Principles of Chromatin Looping. *Cell.* 2014;159(7):1665–80. 10.1016/j.cell.2014.11.021 25497547 PMC5635824

[ref-13] Riley A Prior G : British and Irish Pug Moths – a Guide to their Identification and Biology.2003. 10.1163/9789004475458

[ref-14] Skinner B Wilson D : Colour Identification Guide to the Moths of the British Isles.2009. 10.1163/9789004261020

[ref-15] Uliano-Silva M Nunes JGF Krasheninnikova K : marcelauliano/MitoHiFi: mitohifi_v2.0.2021. 10.5281/zenodo.5205678

