# Supplementary material for: The genome sequence of the double-striped pug, Gymnoscelis rufifasciata (Haworth, 1809)
Source: Wellcome Open Res. 2022 Apr 13;7:135. [Version 1] doi: 10.12688/wellcomeopenres.17790.1 (PMC10403743; doi:10.12688/wellcomeopenres.17790.1)
Supplement: Peer review history

# Reviewer report: Reviewer response for version 1

**DOI:** 10.21956/wellcomeopenres.19690.r62470

**Published:** 2023-09-07

**Authors:** Xue-Yan Li

**Affiliations:** 1 Kunming Institute of Zoology, Chinese Academy of Sciences, Kunming, China

This MS provide a high-quality genome assembly of one moth *Gymnoscelis rufifasciata*, which is very important genome resource of Lepidoptera. However, there are some problems to be carefully checked.

1. How big size for this assembly? In abstract, “The genome sequence is 352 megabases in span.” , but in Genome sequence report “The final assembly has a total length of 462 Mb”.
2. Many sentences, seem a little strange. Part examples were selected from Background and shown as following.

 “A double brooded species, adults are first on the wing in the UK from March to May,” is strange in grammar, please check it.

 “ *Gymnoscelis rufifasciata *is common across western and central Europe, and while it has long been common in southern England, observations in northern England and Scotland have increased dramatically since the year 2000.”  What means “observation”?

Are sufficient details of methods and materials provided to allow replication by others?

Yes

Is the rationale for creating the dataset(s) clearly described?

Yes

Are the datasets clearly presented in a useable and accessible format?

Yes

Are the protocols appropriate and is the work technically sound?

Yes

Reviewer Expertise:

Insect genome

I confirm that I have read this submission and believe that I have an appropriate level of expertise to confirm that it is of an acceptable scientific standard.

# Reviewer report: Reviewer response for version 1

**DOI:** 10.21956/wellcomeopenres.19690.r62475

**Published:** 2023-08-07

**Authors:** Christopher B Cunningham

**Affiliations:** 1 University of Georgia, Athens, Georgia, USA

I think this is a good general resource for the genomics community. The methods seem sound. The article needs a few more details to be reasonably reproducible. Justification or rationale for the work is completely absent.  

 Abstract. There is no justification within the abstract, just results. At least a sentence that this was part of a large survey of insects is needed. And one sentence for its possible value. 

 Keywords. Usually, keywords are required to be in alphabetical order, but check with journal policies. 

 Background. There is no motivation for this study in the introduction. Nothing wrong is said, but the read has no idea why the work was done. Its perfectly fine that the work was done in the context of a large survey, but tell the reader that. Is its expansion north in the UK a problem or likely to become one? The paper is not about its ecology or morphology, but about its genetics. That is not mentioned once. 

 Genome Sequence Report. The BUSCO reference should be in the methods, not results section. 

 Figure 2/3/4/5 titles. These should be informative and tell the reader what you would like them to understand about the data; e.g., Fig 3. Little contamination was found in the final genome assembly. 

 Figure 4. has very little information and can easily be summarized as a sentence in the results. The same is true of Fig 5

 Methods. I assume that the Hi-C library was constructed according to the manufacturer's specifications?

 All parameter setting if they were different from default of each piece of bioinformatic software needs to be specified. Just put a sentence to begin that everything was default unless specified otherwise. What assessment was done to ensure that default was adequate?  

 Table 3 is of little to no value and contains much duplicated information. Just add the version numbers of software used inline at the appropriate places in the methods section. 

 Is the Ethics statement needed? It just says the provider of the sample should meet some standard, but does not actually say that they did in this case. Either drop the statement because there are no legal requirements for insect research beyond basic humane treatment or actually say the standard was met. 

 Add something to the report that contextualize the research for the reader. Is this species the first of a taxon to be assembled, is it an outgroup to some established model species, is it now possible to investigate some interesting aspect of the species biology, etc? This does not to be extensive or highly directed, but there is currently no justification of this work outside of the Data Availability Statement. Even Data Note should contain rationale for the work.

Are sufficient details of methods and materials provided to allow replication by others?

No

Is the rationale for creating the dataset(s) clearly described?

No

Are the datasets clearly presented in a useable and accessible format?

Yes

Are the protocols appropriate and is the work technically sound?

Yes

Reviewer Expertise:

Genome assembly & Annotation, Insects, Molecular Genetics

I confirm that I have read this submission and believe that I have an appropriate level of expertise to confirm that it is of an acceptable scientific standard.

# Reviewer report: Reviewer response for version 1

**DOI:** 10.21956/wellcomeopenres.19690.r60442

**Published:** 2023-08-04

**Authors:** Simon H Martin

**Affiliations:** 1 Institute of Evolutionary Biology, University of Edinburgh, Edinburgh, UK

This note describes a highly complete chromosomal genome assembly for the moth Gymnoscelis rufifasciata.

 The review report from asks whether the rationale for creating the dataset is clearly described. Although no justification is provided within this note, the justification for the DToL project is clearly described elsewhere, and the assemblies, including the one reported here, are already being used as resources in other studies, demonstrating their value.

 The introduction is entirely focused on the UK population. This is understandable given the origin of the sequenced specimen, but it would be helpful to a least mention the full extent of the species’ range.

 One important issue to address is that the abstract says the assembly length is 352 Mb but the main text and figures show that it is 462 Mb.

Are sufficient details of methods and materials provided to allow replication by others?

Yes

Is the rationale for creating the dataset(s) clearly described?

Partly

Are the datasets clearly presented in a useable and accessible format?

Yes

Are the protocols appropriate and is the work technically sound?

Yes

Reviewer Expertise:

Evolutionary genomics, population genetics

I confirm that I have read this submission and believe that I have an appropriate level of expertise to confirm that it is of an acceptable scientific standard.

# Reviewer report: Reviewer response for version 1

**DOI:** 10.21956/wellcomeopenres.19690.r62472

**Published:** 2023-08-02

**Authors:** Henry North

**Affiliations:** 1 University of Cambridge, Cambridge, UK

Boyes *et al.* present a reference genome of *Gymnoscelis rufifasciata*, including both sex chromosomes and the mitochondrial genome. The quality of this assembly is high by all measures of completeness and contiguity. The methods are reported clearly and in detail.

 These data will prove valuable in broader research efforts aimed at understanding chromosomal evolution during the radiation of the Lepidoptera, which requires assemblies from species that span the rich diversity of this order. Pugs such as this species are a familiar occurrence in British light traps and, as the authors point out, *G*. *rufifasciata* is noteworthy for its increased abundance over the past half-century. This assembly permits an investigation of relative population size change deeper in time — when we know what the climate looked like but have no census population size estimates — using coalescent-based demographic inference. The assembly will also be of use in research aimed at understanding the evolution of lepidopteran polyphagy using comparative genomics approaches.

 This publicly available data is a testament to the efforts of the Tree of Life team, and to Douglas’ contribution to the study and public appreciation of the full diversity of the Lepidoptera.

Are sufficient details of methods and materials provided to allow replication by others?

Yes

Is the rationale for creating the dataset(s) clearly described?

Yes

Are the datasets clearly presented in a useable and accessible format?

Yes

Are the protocols appropriate and is the work technically sound?

Yes

Reviewer Expertise:

Population genomics, speciation, adaptation

I confirm that I have read this submission and believe that I have an appropriate level of expertise to confirm that it is of an acceptable scientific standard.
